# Atrial cardiopathy in embolic stroke of undetermined source

**DOI:** 10.1002/brb3.2160

**Published:** 2021-05-04

**Authors:** Jing Chen, Fenglian Gao, Wenhong Liu

**Affiliations:** ^1^ Department of Neurology Peking University Sixth Hospital, Peking University Institute of Mental Health, NHC Key Laboratory of Mental Health (Peking University) National Clinical Research Center for Mental Disorders (Peking University Sixth Hospital) Beijing China; ^2^ Department of Neurology Beijing Shijiitan Hospital, Capital Medical University Beijing China

**Keywords:** atrial cardiopathy, embolic stroke of undetermined source, N‐terminal pro‐B‐type natriuretic peptide, severe left atrial enlargement

## Abstract

**Introduction:**

Atrial cardiopathy is one of the most common potential sources of thromboembolism for embolic stroke of undetermined source (ESUS). The study aims to investigate the incidence of atrial cardiopathy (defined by severe left atrial enlargement (sLAE) or elevated serum N‐terminal pro‐B‐type natriuretic peptide (NT‐proBNP) > 250 pg/ml) in patients with ESUS and compare with other stroke subtypes.

**Methods:**

We retrospectively collected data of 936 consecutive patients with diffusion‐weighted imaging‐confirmed acute ischemic stroke. The incidence of atrial cardiopathy was examined in ESUS, large artery atherosclerosis (LAA), and small vessel disease (SVD) strokes. Clinical characteristics were compared between ESUS patients with atrial cardiopathy (AC‐ESUS) and patients with atrial fibrillation‐induced cardioembolism (AF‐CE) stroke.

**Results:**

245 patients were diagnosed with ESUS, while others were diagnosed with LAA (*n* = 312), SVD (*n* = 258), and AF‐CE (*n* = 121) strokes. The incidence of sLAE in ESUS patients was higher than in LAA or SVD group (5.3% vs. 1.6% and 1.2%, respectively, *p* = .005) and higher than in combined LAA/SVD group (5.3% vs. 1.4%, *p* = .001). The incidence of elevated serum NT‐proBNP in ESUS patients was not statistically different from that in LAA or SVD group. Compared with patients with AF‐CE stroke, AC‐ESUS patients had milder manifestations, had less hemorrhagic transformation, had better short‐term outcome, and had fewer in‐hospital complications.

**Conclusions:**

The incidence of sLAE was higher in ESUS patients than in patients with noncardioembolic strokes. AC‐ESUS was milder when compared to AF‐CE stroke.

## Graphical abstract

The incidence of severe left atrial enlargement is higher in ESUS patients than in patients with noncardioembolic stroke, while there was no difference in the proportion of elevated serum NT‐proBNP between them. ESUS with atrial cardiopathy was a milder form when compared to stroke caused by atrial fibrillation‐induced cardioembolism.

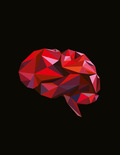

## 1 INTRODUCTION

The term embolic stroke of undetermined source (ESUS) was introduced in 2014 (Hart et al., 2014); this condition accounts for 17% of all ischemic strokes and has a considerable rate of stroke recurrence of 4%–5%/year (Hart et al., 2017; Ntaios, 2020; Ntaios et al., 2015). Therefore, better antithrombotic strategies are urgently needed to decrease the recurrence rate. However, lower stroke recurrence rates were not found from oral anticoagulation than from aspirin in ESUS patients by the two recently completed large trials: NAVIGATE ESUS and RE‐SPECT ESUS (Diener et al., 2019; Hart et al., 2018), which might be explained by the overlap of potential embolic sources, including atrial cardiopathy, covert AF, left ventricular disease, aortic arch plaque, nonstenosing carotid plaques, patent foramen ovale, cardiac valvular disease, and cancer (Ntaios, 2020; Ntaios, Pearce, et al., 2019; Ntaios, Pearce, et al., 2020; Ntaios, Perlepe, Lambrou, et al., 2019; Ntaios, Weng, et al., 2021; Tao et al., 2021). Patients with ESUS caused by emboli from the heart or venous system may benefit from anticoagulation, while patients with ESUS caused by emboli from atherosclerotic plaques respond better to antiplatelet agents. Given this, further studies for appropriately selecting those who respond better to anticoagulation are warranted and urgently needed. In addition, another approach that is used in parallel is to look for patients who have higher likelihood of AF detection. Ntaios et al.'s study revealed that supraventricular extrasystoles on standard 12‐lead electrocardiogram can predict new incident atrial fibrillation after ESUS (Ntaios, Perlepe, et al., 2020), and they have proposed a readily available tool, the AF‐ESUS score, to assist the identification of ESUS patients who have probability of new incident AF, which could potentially support a more personalized strategy (Ntaios, Perlepe, et al., 2021).

Atrial cardiopathy is one of the most common potential sources of thromboembolism. Numerous reports have focused on biomarkers of atrial cardiopathy and their association with the risk of AF detection, the risk of incident ischemic stroke, and the recurrence of ESUS, including increased left atrial size (Benjamin et al., 1995; Jordan et al., 2019; Kamel, Okin, et al., 2019; Perlepe et al., 2020; Yaghi et al., 2015), increased *p*‐wave terminal force in V1 (PTFV1) (Jalini et al., 2019; Kamel et al., 2014; Li et al., 2021), elevated N‐terminal pro‐B‐type natriuretic peptide (NT‐proBNP) (Berntsson et al., 2014; Folsom et al., 2013; Llombart et al., 2015) and so on. And secondary analysis of the NAVIGATE ESUS randomized clinical trial revealed that the risk of ischemic stroke was lower among the rivaroxaban group compared with the aspirin group among the subgroup of patients with a left atrial diameter of more than 4.6 cm (Healey et al., 2019). However, studies on the incidence of elevated serum NT‐proBNP as a biomarker of atrial cardiopathy in ESUS patients are lacking.

The purpose of this study was to investigate the incidence of atrial cardiopathy in patients with ESUS and compare it with that in patients with other stroke subtypes. In addition, we compared the clinical characteristics between ESUS patients with atrial cardiopathy (AC‐ESUS) and patients with atrial fibrillation‐induced cardioembolism (AF‐CE) stroke.

## 2 METHODS

We reviewed the data of consecutive patients with acute ischemic stroke admitted to the stroke unit of Beijing Shijitan Hospital between January 2014 and November 2019. The inclusion criteria were as follows: subjects with acute ischemic stroke confirmed by diffusion‐weighted imaging within 7 days after symptom onset, and subjects undergoing diagnostic workups, including vascular studies (carotid duplex imaging, transcranial Doppler sonography, computed tomography angiography, magnetic resonance angiography, or digital subtraction angiography), transthoracic echocardiogram, and 24 hr Holter monitoring. All cases of acute ischemic stroke that met the inclusion criteria were reviewed by two neurologists. Subjects were first classified by the Trial of Org 10172 in Acute Stroke Treatment (TOAST) criteria (Adams et al., 1993). Among patients with stroke of undetermined etiology, ESUS consensus definition was subsequently applied (Hart et al., 2014). Criteria for diagnosis of ESUS (Hart et al., 2014): stroke detected by MRI that is not lacunar, absence of extracranial or intracranial atherosclerosis causing ≥50% luminal stenosis in arteries supplying the area of ischemia, no major‐risk cardioembolic source of embolism, and no other specific cause of stroke identified (e.g., arteritis, dissection, migraine/vasospasm, and drug misuse). Subjects with AF‐CE stroke were extracted from cardioembolic stroke. The patients classified into ESUS, large artery atherosclerosis (LAA), small vessel disease (SVD), and AF‐CE strokes were included in this study. The exclusion criteria: stroke of other determined etiology; stroke with two or more possible etiologies. The study was approved by the hospital ethics committee.

The following clinical covariates were studied: demographic characteristics, lipid profiles, fasting plasma glucose, HbA1c, homocysteine, serum creatinine, coronary artery disease, and vascular risk factors including hypertension (preexisting diagnosis of hypertension, on medication or blood pressure ≥ 140/90 mmHg), diabetes mellitus (preexisting diagnosis of type I or type II diabetes, on medication or an admitting fasting blood glucose level greater than 126 mg/dl and an HbA1c greater than 6.5% according to the National Glycohemoglobin Standardization Program), smoking (those who had smoked >100 cigarettes in their lifetime and had smoked in the 30 days preceding their stroke), and history of stroke. Initial stroke severity was assessed by the National Institutes of Health Stroke Scale (NIHSS). Short‐term outcome at day 14 or at discharge if earlier was assessed by NIHSS and modified Rankin Scale (mRS). In‐hospital complications were defined as any of the followings: pneumonia, stress ulcer bleeding, or deep venous thrombosis (DVT).

The left atrial diameter and left ventricular end‐diastolic dimension were measured by echocardiographic examination. A left atrial diameter ≥ 47 mm was diagnosed as severe left atrial enlargement (sLAE) based on standard criteria given by the American Society of Echocardiography (Lang et al., 2006). Left ventricular end‐diastolic dimension > 50 mm was diagnosed as left ventricular hypertrophy based on standard criteria given by the Chinese Society of Echocardiography (Li, 2016).

Serum NT‐proBNP was measured in a clinical laboratory using a Cobas e411 analyzer (Roche Diagnostics). The analytic measurement range was 5–35,000 pg/ml. A threshold of 250 pg/ml was chosen to define atrial cardiopathy, as this threshold is associated with a twofold increased risk of recurrent stroke relative to patients with a normal NT‐proBNP level in the Warfarin Aspirin Recurrent Stroke Study (Longstreth et al., 2013), and is used in the ARCADIA randomized trial which is to test the hypothesis that apixaban is superior to aspirin for the prevention of recurrent stroke in subjects with cryptogenic ischemic stroke and atrial cardiopathy (Kamel et al., 2019).

All patients underwent MRI (Ingenia 1.5 or 3.0 T; Philips) within 7 days of admission. Diffusion‐weighted images (DWI), T_1_ and T_2_ weighted images, fluid‐attenuated inversion recovery images, and three‐dimensional time‐of‐flight magnetic resonance angiography images were obtained. Imaging features were defined based on the involvement of infarcts on DWI. Multiple arterial‐territory cerebral infarction (MACI) was defined as more than one acute ischemic lesion in at least two arterial cerebral territories, including the posterior basilar and two anterior carotid territories. Patients with MACI in whom the distribution of the infarcts was related to anatomic variations of the circle of Willis were considered to have a single arterial‐territory cerebral infarction. Hemorrhagic transformation (HT) which is referring to the hemorrhage within the infarct territory or parenchyma hemorrhage outside the infarct zone that was detected later on follow‐up CT or MRI, but not on initial CT (Hacke et al., 1998), detected by routine CT or MRI was obtained, and asymptomatic and symptomatic HTs were both included. Symptomatic HT is defined as hemorrhage that is associated with any decline in neurologic status (National Institute of Neurological & Stroke rt, 1995).

### 2.1 Statistical analysis

Normally distributed continuous variables are presented as mean ± *SD*. Non‐normally distributed continuous variables are presented as median and interquartile range (IQR). Categoric variables are expressed as percentages. For continuous variables, one‐way analysis of variance was used to compare three groups if the group variances were homogeneous or Welch analysis of variance in the presence of heterogeneity; the independent sample *t* test was used to compare two groups for normally distributed variables or the Mann–Whitney U test for non‐normally distributed variables. For categoric variables, the chi‐square or Fisher exact test was performed to compare the groups.

First, we compared clinical characteristics across stroke subtypes. Second, sLAE and elevated serum NT‐proBNP were compared in patients with ESUS, LAA, and SVD strokes. Logistic regression analysis was performed to compare sLAE and atrial cardiopathy between groups after controlling for age, sex, hypertension, diabetes, and creatinine, a prior established as confounding variables for presence of atrial cardiopathy (Jalini et al., 2019), and to compare elevated serum NT‐proBNP between groups after controlling for age, sex, hypertension, diabetes, coronary artery disease, creatinine, and left ventricular hypertrophy, since brain natriuretic peptides secretion increases with these factors (Daniels & Maisel, 2007; Redfield et al., 2002). Finally, clinical and imaging characteristics were compared between ESUS patients with atrial cardiopathy (AC‐ESUS) and patients with AF‐CE stroke. All analyses were performed using SPSS software version 23 (IBM). *p* < .05 was considered statistically significant.

## 3 RESULTS

A total of 936 patients were enrolled, with a mean age of 67.4 ± 12.2 years and 70.6% male. All the patients were classified into ESUS (*n* = 245, 26.2%), LAA (*n* = 312, 33.3%), SVD (*n* = 258, 27.6%), and AF‐CE (*n* = 121, 12.9%) strokes. Serum NT‐proBNP was obtained in 41.2% (*n* = 101) of ESUS, 42.9% (*n* = 134) of LAA, and 35.7% (*n* = 92) of SVD stroke patients.

The clinical characteristics of the ESUS, LAA, and SVD groups are presented in Table 1. The age of the ESUS patients was similar to that of the LAA group, but was higher than that of the SVD group (68.9 ± 12.4 vs. 65.8 ± 11.8, *p* = .005). The proportion of MACI among the ESUS patients was higher than that of the LAA group (odds ratio [OR] = 5.093, 95% confidence interval [CI] = 3.094–8.383, *p *=* *.000). The NIHSS score on admission (2.78 ± 3.92 vs. 3.71 ± 3.74, *p* = .000) and on day 14 (2.39 ± 3.24 vs. 3.68 ± 4.39, *p* = .000), and the mRS score on day 14 (1.84 ± 1.43 vs. 2.40 ± 1.82, *p* = .000) of the ESUS patients were lower than those of the LAA group, but were similar to those in the SVD group. The proportion of pneumonia of the ESUS patients was lower than those of the LAA group (OR = 0.229, 95% CI = 0.114–0.463, *p* = .000), but was similar to those in the SVD group. The proportions of hemorrhagic transformation, stress ulcer bleeding, and deep venous thrombosis among the ESUS patients were similar to those of LAA or SVD group. The distribution of sex, prior stroke, hypertension, diabetes mellitus, cigarette smoking, lipid profiles, fasting plasma glucose, HbA1c, homocysteine, creatinine, and left ventricular hypertrophy did not differ across the three groups.

**TABLE 1:** Baseline characteristics, short‐term outcome, and in‐hospital complications of the ESUS, LAA, and SVD patients

	ESUS (*n* = 245)	LAA (*n* = 312)	SVD (*n* = 258)	*p* value
Baseline characteristics				
Age, mean (*SD*), y	68.9 (12.4)	67.5 (12.2)	65.8 (11.8)	.015
Male, %	69.4	71.5	70.5	.866
Coronary artery disease, %	30.7	29.7	16.7	.000
Prior stroke, %	24.9	27.9	23.6	.488
Hypertension, %	72.2	74.4	69.0	.467
Diabetes mellitus, %	46.9	43.6	47.7	.576
Smoking, %	24.5	33.0	27.9	.081
NIHSS on admission, mean (*SD*)	2.78 (3.92)	3.71 (3.74)	2.32 (2.52)	.000
Total cholesterol, mean (*SD*), mmol/L	4.44 (1.04)	4.45 (1.09)	4.54 (1.14)	.521
HDL, mean (*SD*), mmol/L	1.11 (0.28)	1.07 (0.39)	1.10 (0.26)	.382
LDL, mean (*SD*), mmol/L	2.52 (0.79)	2.64 (1.12)	2.59 (0.94)	.355
Fasting plasma glucose, mean (*SD*), mmol/L	6.75 (2.97)	6.74 (2.44)	6.92 (2.94)	.707
HbA1c, mean (*SD*), %	6.7 (1.6)	6.9 (1.8)	6.8 (1.7)	.476
Homocysteine, mean (*SD*), umol/L	17.7 (12.0)	16.3 (10.0)	18.5 (13.8)	.141
Creatinine, mean (*SD*), μmol/L	76.8 (25.5)	77.9 (28.6)	80.2 (53.7)	.594
Left ventricular hypertrophy, %	18.1	14.5	17.8	.466
MACI, %	30.2	7.7	/	.000
Hemorrhagic transformation, %	1.6	3.8	0.4	.013
Short‐term outcome†				
NIHSS, mean (*SD*)	2.39 (3.24)	3.68 (4.39)	1.93 (2.35)	.000
mRS, mean (*SD*)	1.84 (1.43)	2.40 (1.82)	1.84 (1.35)	.000
In‐hospital complications				
Pneumonia, %	4.1	15.7	4.7	.000
Stress ulcer bleeding, %	4.9	8.6	2.3	.004
Deep venous thrombosis, %	4.5	8.0	2.3	.008

Abbreviations: ESUS, embolic stroke of undetermined source; HbA1c, glycosylated hemoglobin; HDL, high‐density lipoprotein; LAA, large artery atherosclerosis; LDL, low‐density lipoprotein; MACI, multiple arterial‐territory cerebral infarction; mRS, modified Rankin Scale; NIHSS, National Institutes of Health Stroke Scale; SVD, small vessel disease.

^†^ NIHSS and mRS on day 14 or on discharge if earlier.

The incidence of atrial cardiopathy in ESUS, LAA, and SVD groups were presented in Table 2 and Table 3. The incidence of sLAE in ESUS patients was higher than that in LAA, SVD, or the combined LAA/SVD group (5.3% vs. 1.6%, *p *=* *.014, 5.3% vs. 1.2%, *p *=* *.008, and 5.3% vs. 1.4%, *p* = .001, respectively). This was also true for the adjusted analysis as well (OR = 3.216, 95% CI = 1.102–9.392, *p* = .033, OR = 4.014, 95% CI = 1.091–14.747, *p* = .036, and OR = 3.411, 95% CI = 1.352–8.607, *p* = .009, respectively). The incidence of elevated serum NT‐proBNP in ESUS patients was not statistically different from that in LAA or SVD group, while the LAA group had a higher incidence of elevated serum NT‐proBNP than the SVD group (37.3% vs. 12.6%, *p *=* *.000), this was also true for the adjusted analysis as well (OR = 2.104, 95% CI = 1.045–4.237, *p *=* *.037). The incidence of atrial cardiopathy in ESUS patients was higher than that in SVD group (18.4% vs. 10.1%, OR = 2.008, 95% CI = 1.195–3.372, *p *=* *.008), but there was no statistically difference for the adjusted analysis. The incidence of atrial cardiopathy in ESUS patients was higher to that of the LAA or combined LAA/SVD group but without statistically significant difference (18.4% vs. 16.7%, *p *=* *.599, and 18.4% vs. 13.7%, *p *=* *.087, respectively) due to the corresponding percentage of elevated serum NT‐proBNP.

**TABLE 2:** Incidence of atrial cardiopathy in the ESUS versus LAA versus SVD stroke patients

	ESUS	LAA		SVD		*p* value	Adjusted *p* value
	*n* = 245	*n* = 312	^‡^OR (95% CI)	*n* = 258	^‡^OR (95% CI)		
sLAE, *n* (%)	13 (5.3)	5 (1.6)	3.216 (1.102–9.392)	3 (1.2)	4.014 (1.091–14.767)	.005	.034*
NT‐proBNP^†^ ≥ 250 pg/ml, *n* (%)	33 (32.7)	50 (37.3)	1.799 (0.904–3.580)	23 (12.6)	0.788 (0.353–1.730)	.041	.058**
Atrial cardiopathy, *n* (%)	45 (18.4)	52 (16.7)	1.033 (0.640–1.670)	26 (10.1)	1.726 (0.965–3.087)	.021	.080*

Abbreviations: ESUS, embolic stroke of undetermined source; LAA, large artery atherosclerosis; OR (95%CI), odds ratio (95% confidence interval); sLAE, severe left atrial enlargement; SVD, small vessel disease.

^†^ Serum NT‐proBNP was done on 40.1% (327) of patients: 41.2% (101) of ESUS, 42.9% (134) of LAA, and 35.7% (92) of SVD.

^‡^ adjusted odds ratio.

* Logistic regression odds ratio: adjusted for age, sex, hypertension, diabetes, and creatinine.

** Logistic regression odds ratio: adjusted for age, sex, hypertension, diabetes, creatinine, coronary artery disease, and left ventricular hypertrophy.

**TABLE 3:** Incidence of atrial cardiopathy in the ESUS versus LAA/SVD stroke patients

	ESUS (*n* = 245)	LAA/SVD (*n* = 570)	*p* value	^‡^OR (95% CI)	Adjusted *p* value
sLAE, *n* (%)	13 (5.3)	8 (1.4)	.001	3.411 (1.352, 8.607)	.009*
NT‐proBNP^†^ ≥ 250 pg/ml, *n* (%)	33 (32.7)	73 (32.3)	.633	0.725 (0.389, 1.351)	.311**
Atrial cardiopathy, *n* (%)	45 (18.4)	78 (13.7)	.087	1.253 (0.805, 1.950)	.319*

Abbreviations: ESUS, embolic stroke of undetermined source; LAA, large artery atherosclerosis; OR (95%CI), odds ratio (95% confidence interval); sLAE, severe left atrial enlargement; SVD, small vessel disease.

^†^ Serum NT‐proBNP was done on 40.1% (327) of patients: 41.2% (101) of ESUS, and 39.6% (570) of LAA/SVD.

^‡^ adjusted odds ratio.

* Logistic regression odds ratio: adjusted for age, sex, hypertension, diabetes, and creatinine.

** Logistic regression odds ratio: adjusted for age, sex, hypertension, diabetes, creatinine, coronary artery disease, and left ventricular hypertrophy.

We compared the baseline characteristics, short‐term outcome, and in‐hospital complications between AC‐ESUS and AF‐CE stroke patients (Table 4). Our results showed that AC‐ESUS patients had lower NIHSS score on admission and on day14, 1 (0, 4) versus. 4 (2, 9), *p* = .000, and 1 (0, 3) versus. 2 (1, 4), *p* = .039, respectively. Hemorrhagic transformation was less frequent in AC‐ESUS patients than in the AF‐CE group (*p* = .012). Pneumonia and stress ulcer bleeding were less frequent in AC‐ESUS patients than in the AF‐CE group (OR = 0.136, 95% CI = 0.031–0.593, *p* = .002 and OR = 0.232, 95% CI = 0.052–1.036, *p* = .039, respectively). However, no statistically difference was found between the two groups in age of onset, risk factors, the proportion of elevated serum NT‐proBNP, the proportion of sLAE and left ventricular hypertrophy, the proportion of MACI, the mRS score on day 14, and the rate of deep venous thrombosis.

**TABLE 4:** Comparing baseline characteristics, short‐term outcome, and in‐hospital complications of the AC‐ESUS and AF‐CE stroke patients

	AC‐ESUS (*n* = 43)	AF‐CE (*n* = 121)	t or Z or OR (95%CI)	*p* value
Baseline characteristics				
Age, median (IQR), y	81 (73,84)	79 (71, 84)	−0.636	.525
Male, %	58.1	52.1	1.237 (0.612–2.499)	.493
Coronary artery disease, %	51.2	44.9	1.149 (0.538–2.455)	.460
Prior stroke, %	30.2	33.9	0.846 (0.399–1.793)	.664
Hypertension, %	79.1	78.5	1.034 (0.440–2.427)	.939
Diabetes mellitus, %	51.2	40.5	1.344 (0.669–2.700)	.225
Cigarettes smoking, %	14.0	9.9	0.992 (0.364–2.706)	.467
NIHSS on admission, median (IQR)	1 (0, 4)	4 (2, 9)	−3.496	.000
Total cholesterol, median (IQR), mmol/L	4.31 (3.56, 5.15)	4.00 (3.49, 4.72)	−1.591	.112
HDL, median (IQR), mmol/L	1.10 (0.99, 1.26)	1.17 (1.02, 1.34)	−1.138	.255
LDL, median (IQR), mmol/L	2.61 (1.84, 3.07)	2.18 (1.75, 2.70)	−1.465	.143
Fasting plasma glucose, median (IQR), mmol/L	5.92 (4.93, 7.40)	6.00 (5.03, 7.55)	−0.247	.805
HbA1c, median (IQR), %	6.50 (5.60, 7.85)	6.05 (5.70, 6.88)	−0.667	.504
Homocysteine, median (IQR), umol/L	16.1 (12.1, 19.1)	14.3 (11.8, 17.8)	−1.133	.257
Creatinine, median (IQR), μmol/L	78.5 (59.5, 98.25)	70.0 (57.0, 88.0)	−1.567	.117
NT‐proBNP^†^ ≥ 250 pg/ml, %	84.2	88.4	0.702 (0.235–2.094)	.524
sLAE, %	27.9	24.0	1.228 (0.559–2.696)	.608
Left ventricular hypertrophy, %	23.3	17.8	1.081 (0.414–2.824)	.874
MACI, %	27.9	22.3	1.348 (0.610–2.975)	.459
Hemorrhagic transformation, %	0	13.2	/	.012
Short‐term outcome^‡^				
NIHSS, median (IQR)	1 (0, 3)	2 (1, 4)	−2.069	.039
mRS, median (IQR)	2 (1, 3)	2 (1, 4)	−1.438	.138
In‐hospital complications				
Pneumonia, %	4.7	26.4	0.136 (0.031–0.593)	.002
Stress ulcer bleeding, %	4.7	17.4	0.232 (0.052–1.036)	.039
Deep venous thrombosis, %	4.7	12.4	0.345 (0.075–1.574)	.152

Abbreviations: AC‐ESUS, embolic stroke of undetermined source associated with atrial cardiopathy; AF‐CE, atrial fibrillation‐induced cardioembolism; HbA1c, glycosylated hemoglobin; HDL, high‐density lipoprotein; LDL, low‐density lipoprotein; MACI, multiple arterial‐territory cerebral infarction; mRS, modified Rankin Scale; NIHSS, National Institutes of Health Stroke Scale; OR (95%CI), odds ratio (95% confidence interval).

^†^ Serum NT‐proBNP was done on 75% (123) of patients: 88.4% (38) of AC‐ESUS, and 71.1% (86) of AF‐CE.

^‡^ NIHSS and mRS on day 14 or on discharge if earlier.

## 4 DISCUSSION

In our study, we observed that the incidence of sLAE was higher in ESUS patients than in patients with noncardioembolic strokes, while there was no difference in the proportion of elevated serum NT‐proBNP between ESUS patients and patients with noncardioembolic strokes. AC‐ESUS was a milder form when compared to AF‐CE stroke, while they had many similarities.

sLAE, as a marker of atrial cardiopathy, occurred more frequently in ESUS than in noncardioembolic strokes, which implied that atrial cardiopathy may be a possible risk factor for ESUS. Studies have shown that the histologic clot composition in cryptogenic stroke patients is more similar to that in cardioembolic stroke patients (high fibrin, low red blood cell content) than to that in stroke patients with noncardioembolic etiologies (high erythrocyte content) (Boeckh‐Behrens et al., 2016; Sporns et al., 2017), which supports the hypothesis that ESUS pathogenesis is cardiac in origin; thus, covert paroxysmal atrial fibrillation (AF) is thought to be a leading cause of stroke in ESUS. However, further studies indicated that covert atrial fibrillation as an ESUS etiology appears to be less important than initially thought. A long‐term follow‐up study found that only 30% of cryptogenic stroke patients manifested any AF even after 3 years of continuous heart rhythm monitoring via an implantable loop recorder (Sanna et al., 2014). Studies have shown a lack of temporal relationship between embolic events and AF. The majority of embolic events (stroke or systemic embolism) do not occur proximal to recent episodes of atrial tachycardia or AF, as shown in patients with implantable cardiac monitoring devices in the ASSERT and TRENDS studies (Brambatti et al., 2014; Daoud et al., 2011). Ntaios et al.'s study revealed that stroke severity of ESUS patients who were diagnosed with AF during follow‐up is similar to those who were not, which also questions the causal relationship between ESUS and AF detected during the follow‐up (Ntaios et al., 2016). A meta‐analysis of eight randomized clinical trials found no evidence of any reduction in stroke risk with rhythm‐control strategies versus rate‐control strategies (Martin et al., 2015). Our results, along with other studies, suggest that thrombus formation in the left atrium may not be necessarily a result of AF dysrhythmia but rather a result of pathologic changes within the left atrium, which is called atrial cardiopathy. Most likely, the major value of the concept of atrial cardiopathy is that it provides an opportunity for improved prevention of stroke. It may be that patients with atrial cardiopathy can be optimally treated with anticoagulants, just as is currently the standard practice for patients with AF.

In our study, atrial cardiopathy was defined by the presence of sLAE or elevated serum NT‐proBNP. Left atrial enlargement was associated with the risk of AF detection, the risk of incident ischemic stroke, and the recurrence of ESUS (Jordan et al., 2019; Kamel, Okin, et al., 2019; Perlepe et al., 2020; Yaghi et al., 2015). We found that the incidence of sLAE in ESUS patients was in accordance with other published studies and higher than that in LAA or SVD group, even after adjusting for age, sex, hypertension, diabetes, and creatinine, which was consistent with Kamel et al.'s result (Yaghi et al., 2016). However, there was no difference in the frequency of sLAE between ESUS, LAA, and SVD groups in Shirin et al.'s study (Jalini et al., 2019), so prospective multicenter studies are needed to confirm our results.

Studies had shown an association between elevated serum NT‐proBNP and ischemic stroke, particularly of embolic stroke subtype (Berntsson et al., 2014; Folsom et al., 2013; Llombart et al., 2015). A post hoc analysis of the WARSS trial showed a reduction in the risk of stroke or death among those assigned warfarin rather than aspirin among the 5% of patients with the highest levels of NT‐proBNP (Longstreth et al., 2013). All of these findings imply that elevated serum NT‐proBNP is associated with cardioembolism. However, the proportion of elevated serum NT‐proBNP in ESUS patients did not differ from that in the LAA or SVD group in our study, possible explanations: brain natriuretic peptide are produced both in atria and in ventricles in response to increased transmural wall stress, which is critical to cardiorenal regulation, so the increase in serum NT‐proBNP is affected by many factors, including age, sex, left ventricle dysfunction, renal failure, and so on (Daniels & Maisel, 2007; Redfield et al., 2002); serum NT‐proBNP in our study was not tested in the same time period from stroke onset; serum NT‐proBNP levels were measured in less than half of all patients, as part of routine practice and physician judgment in the real world. The fact that there was no difference in the proportion of elevated serum NT‐proBNP across the three groups in our study does not mean that elevated serum NT‐proBNP cannot be used as a biomarker of atrial cardiopathy. In practice, when we meet ESUS patients with elevated serum NT‐proBNP, a more individualized approach to patients should be adopted to determine whether it is indicative of atrial cardiopathy. The frequency of elevated serum NT‐proBNP in ESUS patients in our study is somewhat lower than that previously reported. We identified elevated serum NT‐proBNP in 32.7% of our ESUS patients, while others reported elevated serum NT‐proBNP levels in up to 49% of cryptogenic stroke (Yaghi et al., 2016). However, comparisons with previous studies are difficult since inclusion criteria vary.

There was a higher prevalence of MACI among ESUS than among LAA patients. A prior study found that 39% of ESUS patients had multiple infarcts on diffusion‐weighted imaging, and multiple infarcts were more frequent among patients with recurrent vascular events (Ueno et al., 2016). However, there are few data on the prevalence of MACI among ESUS patients compared with other stroke subtypes. The higher prevalence of MACI among ESUS patients may be related to the underlying pathophysiologies, including cardioembolism, artery to artery embolism from the aortic arch atheroma, paradoxical embolism from the venous system, and hypercoagulability caused by occult cancer or autoimmune disease. So cardiac evaluation will be beneficial to the prevention of stroke and stroke recurrence.

AC‐ESUS was a milder form of stroke when compared to AF‐CE stroke. For AC‐ESUS patients, it is possible that this condition represents a milder form of atrial dysfunction, which, as it advances, produces the dysrhythmia of AF. In this milder form, stroke may be caused by a smaller clot, thus resulting in a smaller infarct volume, a lower risk of reperfusion injury and, consequently, less hemorrhagic transformation (Jalini et al., 2019). However, AC‐ESUS and AF‐CE stroke patients had many similarities as mentioned above. The differences and similarities of these two groups support the ideas that atrial cardiopathy can cause cardioembolic stroke without AF and AF is one manifestation of atrial cardiopathy.

Our study had several limitations. First, it was a retrospective, single‐center study. Second, our ESUS patients generally did not undergo more than 24 hr of heart rhythm monitoring, which may underestimate the incidence of AF, since continuous ECG monitoring for at least 72 hr with an increased proportion of AF detection was recommended for patients with ischemic stroke or TIA (Schnabel et al., 2019). Third, the left atrial diameter was used as a measure of sLAE, while investigations have shown left atrial volume index to be a better marker of atrial cardiopathy (Tan et al., 2020). This information was not available in our study. Fourth, the serum NT‐proBNP levels were measured in less than half of all patients and were only measured on admission, not serially measurements. We are uncertain if the NT‐proBNP levels may have risen acutely during an acute ischemic stroke event, and hence may not be a true reflection of atrial cardiopathy. Fifth, other markers of atrial cardiopathy including Electrocardiographic P‐wave markers were not explored in this study. Investigations have shown the increased P‐terminal force in the precordial lead V1 (PTFV1) was associated with AF detection and had higher prevalence in ESUS than LAA, SVD, or combined LAA/SVD patients (Jalini et al., 2019; Li et al., 2021). Sixth, supracardiac atherosclerosis including the atherosclerotic plaque in the carotid, vertebrobasilar, and intracranial arteries, or the aortic arch, which is another important embolic source in ESUS (Ameriso et al., 2020; Kamel et al., 2020  ; Ntaios, Pearce, et al., 2019; Ntaios, Perlepe, Sirimarco, et al., 2019; Ntaios, Sagris, et al., 2021; Ntaios, Swaminathan, et al., 2019; Ntaios et al., 2020), was not investigated in our study. Nearly half of ESUS patients have ≥2 diseases which could be considered as the cause of stroke (Ntaios, Perlepe, Lambrou, et al., 2019), so no available data on supracardiac atherosclerosis in ESUS patients may influence our results.

## 5 CONCLUSIONS

In our study, the incidence of sLAE is higher in ESUS patients than in patients with noncardioembolic strokes, which is a reliable biomarker of atrial cardiopathy. However, the elevated serum NT‐proBNP, as a biomarker of atrial cardiopathy, should be used individually. AC‐ESUS was a milder form when compared to AF‐CE stroke, while they had many similarities. Our results support the concept that atrial cardiopathy is an important embolic source of ESUS. Therefore, for ESUS patients with atrial cardiopathy, anticoagulation may be a better choice for preventing stroke recurrence. The ongoing ARCADIA research trial, which is testing the hypothesis that the direct oral anticoagulant apixaban is superior to aspirin for the prevention of recurrent stroke in ESUS patients with atrial cardiopathy, appears promising (Kamel et al., 2019).

## CONFLICT OF INTEREST

None declared.

## AUTHOR CONTRIBUTIONS

All authors contributed to the study conception or design and to the interpretation of the results. All authors read and revised the manuscript and approved the final manuscript to be submitted.

### PEER REVIEW

The peer review history for this article is available at https://publons.com/publon/10.1002/brb3.2160.

## Data Availability

The data that support the findings of this study are available on request from the corresponding author. The data are not publicly available due to privacy or ethical restrictions.
